# Role of microRNAs in Hemophilia and Thrombosis in Humans

**DOI:** 10.3390/ijms21103598

**Published:** 2020-05-20

**Authors:** Katarzyna I. Jankowska, Zuben E. Sauna, Chintamani D. Atreya

**Affiliations:** 1 OBRR/DBCD/LCH in the Center for Biologics Evaluation and Research, US Food and Drug Administration, Silver Spring, MD 20993, USA; katazyna.jankowska@fda.hhs.gov; 2 OTAT/DPPT/HB in the Center for Biologics Evaluation and Research, US Food and Drug Administration, Silver Spring, MD 20993, USA; zuben.sauna@fda.hhs.gov

**Keywords:** microRNAs (miRNAs), thrombosis, bleeding disorders, coagulation factors, hemophilia

## Abstract

MicroRNAs (miRNA) play an important role in gene expression at the posttranscriptional level by targeting the untranslated regions of messenger RNA (mRNAs). These small RNAs have been shown to control cellular physiological processes including cell differentiation and proliferation. Dysregulation of miRNAs have been associated with numerous diseases. In the past few years miRNAs have emerged as potential biopharmaceuticals and the first miRNA-based therapies have entered clinical trials. Our recent studies suggest that miRNAs may also play an important role in the pathology of genetic diseases that are currently considered to be solely due to mutations in the coding sequence. For instance, among hemophilia A patients there exist a small subset, with normal wildtype genes; i.e., lacking in mutations in the coding and non-coding regions of the *F8* gene. Similarly, in many patients with missense mutations in the *F8* gene, the genetic defect does not fully explain the severity of the disease. Dysregulation of miRNAs that target mRNAs encoding coagulation factors have been shown to disturb gene expression. Alterations in protein levels involved in the coagulation cascade mediated by miRNAs could lead to bleeding disorders or thrombosis. This review summarizes current knowledge on the role of miRNAs in hemophilia and thrombosis. Recognizing and understanding the functions of miRNAs by identifying their targets is important in identifying their roles in health and diseases. Successful basic research may result in the development and improvement of tools for diagnosis, risk evaluation or even new treatment strategies.

## 1. Introduction

According to the National Hemophilia Foundation, bleeding disorders are a group of disorders that share the inability to form a proper blood clot; they are characterized by extended bleeding after injury, surgery, trauma or menstruation. The human body produces 13 clotting factors also known as coagulation factors and any deficiency in any one of them can lead to either a minor, moderate or severe bleeding disorder (https://www.hemophilia.org/Bleeding-Disorders/What-is-a-Bleeding-Disorder). 

Conversely, a blood clot could result in blood vessels due to high levels of some of the clotting factors (coagulation factors VII, VIII, IX, XI and von Willebrand factor), which is known as thrombosis, a term originated from Greek, meaning a lump or clump (https://www.medicinenet.com/script/main/art.asp?articlekey=25023). 

Both deficiency of and excessive production of clotting factors can result in a disease pathology. Maintenance of homeostasis vis-à-vis clotting factors is thus critical for avoiding pathologies. Several cellular regulatory mechanisms, operating at several levels are involved in the regulation of gene expression. For example, regulation can occur at the gene transcriptional level (mRNA size, polyadenylation and copy number), posttranscriptional level (translational interference) and posttranslational level (phosphorylation, glycosylation, etc.). 

In the last few decades, microRNA (miRNA)-mediated control of mRNA expression has emerged as a key biological mechanism regulating gene expression. The miRNAs are small noncoding RNAs that bind to the 3′ untranslated region (UTR) of target mRNAs and through a complex process affect gene expression [1,2,3]. miRNAs exert their influence on gene expression by either terminating [4,5], or fine-tuning [6,7]. 

In the case of the rare bleeding disorder hemophilia A (HA), miRNAs have been shown to directly target and down-regulate the *F8* gene that codes for Factor VIII (FVIII) [8,9]. 

The pathogenesis of thrombosis is more complex and can be triggered by numerous inherited or environmental factors (or a combination of both) leading to arterial or venous occlusion. 

The cumulative effect is abnormalities in the vessel wall (e.g., atherosclerosis), blood flow or blood coagulation, which in turn may lead to alterations in platelet function, levels of coagulation factors or fibrinolysis. In addition, thrombosis may be triggered by metabolic or hormonal factors as well as by endothelial dysfunction and inflammation [10]. Among the many risk factors for thrombosis, levels of FVIII that are >1.5 times the normal plasma levels (0.5–1.5) have shown a strong association with venous thrombosis [11].

## 2. Bleeding Disorders and miRNAs 

A growing body of research indicates that miRNAs play a role as modulators of the hemostatic system by direct or indirect interaction with the mRNAs that encode proteins involved in coagulation. Dysregulation of these miRNAs can lead to expression of coagulation proteins that are outside the relatively narrow range observed in healthy individuals which leads to either bleeding disorders or thrombosis (Table 1).

### 2.1. Hemophilia

Bleeding disorders such as HA and hemophilia B (HB), are X-linked genetic diseases cause by deficiency of coagulation factor VIII and coagulation factor (FIX), mainly due to genetic perturbations or variations in the *F8* and *F9* genes [34]. In a small subset of screened hemophilia patients, *F8* and *F9* gene do not exhibit any mutations. In approximately 0.6% of severe HA, 2.9% of mild-moderate HA patients and 1.1% of mild to moderate HB patients no variants were identified. Nonetheless these individuals showed lower levels of FVIII or FIX consistent with the severity of the disease, suggesting that the expression of these coagulation factors is controlled by mechanisms besides genetic mutations in *F8* and *F9* genes in the disease manifestation.

#### 2.1.1. Hemophilia A

While in most cases HA is associated with mutations in the *F8* gene, several lines of evidence indicate a role of miRNAs in FVIII deficiency. We carried out a microarray analysis of blood samples from 15 HA patients with or without inhibitors (inhibitory anti-FVIII antibodies) to test the hypothesis that dysregulation of miRNAs that control immune response genes contribute to inhibitor development in some HA patients; we discovered that upregulation of miR-1246, miR-4521 and miR-181d in HA patients [9]. We also demonstrated that miR-1246 has a potential target site in 3′UTR of FVIII as predicted by Target Scan and can modulate *F8* expression in lymphoblastoid cells that endogenously express FVIII. Thus, we inadvertently discovered that impeded regulation of FVIII expression could contribute to the HA phenotype. A miRNA mediated inhibition of FVIII leading to HA is best evaluated in the ultra-rare patients where mutations in the coding and non-coding *F8* sequences are not a confounding factor. 

Next generation sequencing analysis of blood samples from severe and mild HA patients with no genetic defect in coding or non-coding regions revealed a group of 8 miRNAs significantly dysregulated compare to healthy donors; two miRNAs, miR-128-3p and let-7i-5p were down-regulated and 6 miRNAs (miR-144-5p, miR-374b-5p, miR-30c-5p, miR-6803-3p, miR-15b-3p and miR-483-3p) were up-regulated in HA patients; from this pool, miR-30c and miR-374b were demonstrated to target 3′UTR of FVIII [8]. Both, miR-30c and miR-374b were shown to target and regulate expression of the *F8* gene in lymphoblastoid cells and, downregulate FVIII protein levels in lymphoblastoid cells and the Human Umbilical Vein Endothelial Cells (HUVECs). Importantly, inhibition of endogenous miR-30c in the HUVEC cell line resulted in a significant increase of endogenous FVIII. These studies confirmed a role for miRNAs in regulating FVIII levels and indicated that dysregulation of miRNAs could contribute to FVIII deficiency. The finding that controlling expression of miRNAs can modulate FVIII protein levels in cell is important as it suggests a potential therapeutic strategy for the clinical use of miRNAs [35]. 

It is also possible that genetic variations in miRNA target sites that disrupt the formation of the miRNA-mRNA duplex can contribute to FVIII deficiency. Consistent with this postulate; several mutations in the 3′UTR of *F8* have been associated with HA [36] and occur in ~0.1% of patients [34]. Intriguingly, mutation in *F8* 3′UTR associated with HA are very close to the target sites for miR-26a-5p, miR-26b-5p and miR-1297 [21]. 

#### 2.1.2. Hemophilia B

The role of miRNA in hemophilia B has not yet been extensively studied. It is however interesting to note that 3′UTR polymorphisms of coagulation factor IX are more frequent than those identified in FVIII and occur in 1.4% of severe and 1.1% mild-moderate HB patients [34]. Thus, the postulate that FIX levels are affected in some HB patients via miRNA dysregulation warrants further investigation. 

In addition, recent studies show that miR-128 and miR-125 may play a role in the expression of a *F9* gene variant with a nonsense mutation (E7a (nt 34 G > T in exon 7) and E7b (nt 52 G > T in exon 7). It has been proposed that the miRNAs suppress the nonsense-mediated mRNA decay (NMD) pathway which degrades the mRNAs containing the premature termination codon (PTC) [22]. These miRNAs provide a potential therapeutic avenue for HB patients with nonsense mutations who represent ~24% of severe HB patients [34]. 

#### 2.1.3. Hemophilia C

Factor XI (FXI) deficiency, known also as hemophilia C can be caused by more than 220 mutations in the *F11* gene [37]. The deficiency of FXI does not always cause spontaneous bleeding, but rather increases the risk of bleeding during hemostatic events [38]. The mechanism of FXI regulation is complex and still not fully understood, however a recent study suggests that *F11* gene expression is regulated by miRNAs [39]. Overexpression or suppression of miR-181a in the liver cell line, HepG2 resulted on dysregulation of FXI. The direct interaction of this miRNA with *F11* 3′UTR was confirmed by using in silico algorithms and in an in vitro study using the luciferase assay. Moreover, *F11* mRNA levels were inversely and significantly correlated with miR-181a-5p levels in 114 healthy livers [39]. Consequently, up-regulation of miR-181a may cause a decrease in FXI expression and FXI deficiency in the plasma. An additional study on the genetic regulation of FXI predicted 11 miRNAs including miR-145 and miR-181a as potential post-transcriptional regulators of FXI [24]. Further evaluation of these two miRNAs via luciferase assay confirmed the direct interaction of miR-145 and miR-181a with *F11* 3′UTR.

### 2.2. Von Willebrand Disease 

Von Willebrand disease (VWD) caused by genetic mutations in the Von Willebrand factor (VWF) gene is the most common inherited hemostatic disorder and associated with over 560 mutations and 217 polymorphisms in *VWF* gene [19]. Polymorphisms in the 5′ and 3′ UTR regions of the *VWF* gene have been reported but the effect of miRNAs on different variants has not been studied. Interestingly, a study on blood samples from 115 diabetic patients and 112 healthy donors identified a correlation between high glucose level, elevated levels of mature VWF and lower expression of miR-24 compared to healthy controls [32]. The direct association of miR-24 with the *VWR* gene was confirmed by prediction analysis and reporter gene assays using a luciferase vector linked to either a wild-type *VWF* 3′UTR or the mutated miR-24 binding site. Finally, studies using a diabetic mouse models and human and mouse endothelial cells further confirmed that suppression of miR-24 increases the expression of VWF while overexpression of miR-24 decreases WVF expression. 

An additional study found that miR-24 associates with the 3′UTR of VWF in osteosarcoma (OS) tissues collected from 84 patients [33]. Decreased expression of miR-24 in human OS tissues was closely associated with tumor metastasis and OS cell progression. 

If these studies are replicated and the mechanistic role of VWF as a biomarker for diabetes and OS are elucidated; modulating miRNA levels offers an alternative clinical strategy for the clinical management of these diseases. As these two studies have shown that miR-24 regulates expression, maturation and secretion of VWF, additional studies to evaluate the role of miRNAs in control on VWF expression and in VWD are warranted. 

## 3. Thrombosis and miRNA 

Thrombosis, that can occur in the veins or the arteries is a leading cause of death worldwide [40]. This complex disease is associated with both environmental and genetic risk factors. In addition, the role of miRNA in thrombosis has been recently accentuated (Table 2).

### 3.1. Venous Thromboembolism

Venous thrombosis that includes both deep vein thrombosis (DVT) and pulmonary embolism (PE) is one of the major causes of cardiovascular disease. DVT is a serious condition that occurs when a blood clot forms in a vein located deep inside a patient; it typically forms in the foot, ankle and leg as well as the arm. While it is commonly accepted that the venous thrombosis is associated with numerous environmental factors genetic perturbations that cause loss of function in antithrombin, protein S, protein C or high level of coagulation factors have also been associated with clot formation in veins [70]. The evaluation of plasma from 39 venous thromboembolism (VTE) patients with recurrent VTE compared to 39 control non-recurrent VTE patients found that elevated level of: miR-15b-5p, miR-222-3p, miR-26b-5p, miR-532-5p, miR-21-5p and miR-30c-5p and lower levels of miR-106a-5p, miR-197-3p, miR-652-3p, miR-361-5p, miR-27b-3p and miR-103a-3p were significantly associated with risk of VTE recurrence [60]. Another study on 20 patients with a history of unprovoked VTE showed an increase in expression of miR-10b-5p, miR-320a/b, miR-424-5p and miR-423-5p, and a decrease in expression of miR-103a-3p, miR-191-5p, miR-301a-3p and 199b-3p in patient plasmas compare to healthy controls [59]. Furthermore, a meta-analysis that included a total of 1057 individuals suggested that miR-1233, miR-134, miR-145, miR483-3p, miR-532 and miR-195 may serve as VTE diagnostic biomarkers [61]. 

#### 3.1.1. Deep Vein Thrombosis 

Numerous reports suggest a role for miRNAs in the pathogenesis of venous thrombo-embolism and some of these studies have found a strong correlation between differential miRNAs expression and the symptoms of venous thrombosis [71,72]. The majority of miRNAs associated with deep vein thrombosis has been shown to be expressed by endothelial cells (ECs) and are associated with the normal function(s) of ECs [44]. The process by which endothelial progenitor cells (EPCs), i.e., bone marrow-derived cells that have the capacity to migrate to the peripheral circulation and to differentiate into mature endothelial cells, have been shown to be regulated by numerous miRNAs. Dysregulation of these miRNA results in dis-functional endothelial progenitor cells that may trigger deep vein thrombosis [73]. 

Mir-483-3p that has been upregulated in DVT patients has been shown to control the expression of serum response factor (SRF). Decreased levels of SRF results in reduced migration and tube formation and increased apoptosis of EPCs [52]. The miR-21 was shown to be directly associated with the 3′UTR of *FASL* which encodes the Fas ligand, a transmembrane receptor that is known to induce apoptosis. Moreover, lower expression level of miR-21 in DVT patients was associated with an increase of recurrent DVT and post thrombotic syndrome (PTS) [48]. The proliferation of EPCs has also been shown to be associated with miR-150 which controls expression of the SRC kinase signaling inhibitor 1 (SRCIN1) [43]. Overexpression of miR-150 and concomitant decreased in the levels of SRCIN1 resulted in thrombus resolution in a murine model of venous thrombosis. and promoted angiogenesis and proliferation of EPCs. 

Furthermore, it has been shown that progression of endothelial progenitor cells can be controlled by miR-126 by directly targeting the *PIK3R2* gene whose gene product affects PI3K/Akt signaling. Overexpression of mir-126 enhanced the migratory and tubulogenic properties of EPCs in vitro and promoted EPCs’ homing and thrombus resolution in vivo [41]. A similar effect was observed in EPCs cells upon overexpression of miR-205 that has a target site on the 3′UTR of the *PTEN* gene. Regulation of the PTEN gene product can modulate the Akt/autophagy pathway and MMP2 expression, which are essential for EPC function and DVT recanalization and resolution [47]. 

The inhibition of miR-195 in EPCs promoted cell proliferation, mitigation and angiogenesis by targeting the GABA type A receptor associated protein like 1 (GABARAPL1) [45]. While in another study, the high level of miR-195-5p in the blood of DVT patients has been shown to be directly associated with low expression of B-cell lymphoma 2 (Bcl-2). This miRNA may thus play a role in the development of DVT by regulating the apoptosis of vascular endothelial cells [44]. 

In addition to common risk factors, the studies have shown that deep vein thrombosis can be triggered by alteration of inflammatory processes including cytokines, chemokines and different types of leukocytes. It has been shown that miRNAs are the key gene regulators controlling inflammation [74]. Elevated levels of interleukin-6 (IL-6), that stimulate thrombus formation in veins, has been associated with low expression of miR-338-5 in peripheral blood mononuclear cells (PBMCs) obtained from patients with DVT [51]. Evaluation of plasma from 81 patients with lower-extremity deep vein thrombosis showed a decrease in expression of miR-103-3p and increased expression of chemokine C-X-C motif ligand 12 (CXCL12) that has a target site for this miRNA [26]. miRNA assessment in PBMCs from 45 patients with DVT and healthy donors showed down-regulation of miR-26a. MiR-26a directly targets and suppresses the expression of protein kinase C δ(PRKCD), which significantly reduces the levels of chemokine C-C motif ligand (CCL)2 and thus inhibits activation of the nuclear factor kappa B (NF-κB) signaling pathway [49]. 

The use of miRNAs as reliable and predictive biomarkers to detect the early signs of deep vein thrombosis has been extensively evaluated. Evaluation of serum from 18 patients with deep vein thrombosis showed up-regulation of miR-582, miR-195 and miR-532 compared to healthy controls [46]. MiRNAs analysis of 12 DVT patients and 12 healthy donors revealed 13 miRNAs dysregulated in DVT patients. An investigation of 238 DVT patients showed significant upregulation of miR-424-5p and downregulation of miR-136-5p in patient plasma. The plasma level of miR-424-5p was associated with both D-dimer and APC-PCI complex levels [42]. D-dimer, a fiber degradation protein fragment released into blood after blot clot, is elevated in patients with thrombotic disorder and used as a biomarker to identify DVT. The studies on 30 DVT patients, 30 post-thrombotic syndrome patients and 30 healthy volunteers unveil significant dysregulation of miR-320a/b in DVT compare to both PTS or healthy groups [50].

#### 3.1.2. Pulmonary Embolism (PE)

Evaluation of miR-27a/b expression in 78 patients with acute pulmonary embolism (APE) and 70 healthy donors displayed significantly higher expression of this miRNA in APE patients [57]. It has been shown that miR-27a/b target and regulate the tissue factor pathway inhibitor (TFPI) in endothelial cells thus indirectly regulating tissue factor (TF) and blood coagulation [31,75]. Evaluation of ten chronic thromboembolic pulmonary hypertension (CTEPH) patients revealed upregulation of 17 miRNAs. Among them, let-17b that was downregulated in plasma of CTEPH patients has been shown to directly target endothelin-1 (ET-1) and cause dysfunction of pulmonary arterial endothelial cells [53]. Another study on 32 APE patients and 32 healthy donors showed significant upregulation of miR-134 in the plasma of APE patients [55]. Studies on a group of 60 patients with acute APE and 50 healthy volunteers, showed a high level of miR-221 in APE samples compared to controls [56]. High level of miR-28 was observed in 37 PE patients compared with normal controls [58]. Finally, miR-1233 has been shown to be selectively upregulated in acute PE compared to patients with chronic PE and healthy donors [54]. Additional studies are needed to distinguish the precise regulatory role of these miRNAs in PE and to verify which of these miRNAs may serve specific diagnostic predictors of APE.

### 3.2. Arterial Thrombosis

Arterial thrombosis is caused by a rupture of atherosclerotic plaque (rich in platelets) formed after accumulation of the lipid and lipid-laden macrophages in the artery wall [40]. The pathogenesis of arterial thrombosis is complex and is associated with numerous genetic and environmental factors related to atherosclerosis and thrombosis, as well as the interaction of the two [76]. Several reports have highlighted the role of miRNA in atherosclerosis as they fine-tune the expression of proteins involved in the progression, modulation and regulation of every stage of atherosclerosis including lipid homeostasis, signaling pathways and endothelial function [77,78,79]. For example, it has been suggested that the process of atherosclerotic plaque rupture may be controlled by miR-223-mediated suppression of tissue factor [30].

Platelet number and activation is linked with arterial thrombosis and coronary artery disease [80,81,82,83]. A study on patients with essential thrombocythemia (ET) who had elevated platelet counts revealed downregulation of 9 miRNAs: miR-10a, miR-28, miR-126, miR-155, miR-221, miR-222, miR-223 and miR-431 as well as upregulation of miR-9 and miR-490 in ET patients compared to healthy controls [62]. Moreover, increased mir-223 level has been also associated with lipid metabolism and high intracellular cholesterol levels that can trigger lipid metabolism-related disorders including liver steatosis and atherosclerosis [84]. 

In other studies, the high level of miR-126 detected in patients with coronary artery disease (CAD) was associated with high low-density lipoprotein (LDL) cholesterol levels. Interestingly, low levels of miR-126 were associated with a high level of LDL in patients who had risk factors for CAD but did not have angiographically significant CAD [66]. miR-126 has been shown to play a role in arterial remodeling by crosstalk with miR-221/222 in endothelial cells [65,85]. By binding to vascular cell adhesion molecule-1 (VCAM-1) miR-126 can modulate adhesion of leucocytes to endothelial cells and thus play an anti-inflammatory role. Moreover, miR-126 that is released by microparticles during EC apoptosis is linked to atherosclerosis through regulation of CXCL12. Overall, this study suggests that miR-126 can serve as a marker atherothrombotic vascular diseases [64]. Mir-19b that has been shown to target tissue factor in patients with unstable angina may also play an anti-thrombotic role [29]. An association of mir-19b with cholesterol metabolism and atherosclerotic diseases has been demonstrated [86]. Mir-19b directly targeted adenosine triphosphate (ATP)-binding cassette transporter A1 and its overexpression increase plaque size and lipid content. 

Role of other miRNA: miR-181b in regulation of TF and arterial thrombosis was also highlighted [28]. MiR-181b indirectly regulated TF by targeting thrombin-activated NF-κB signaling.

Moreover, suppression of miR-124a and miR-125a and overexpression of miR-146a and miR-155 in monocytes was associated with atherothrombosis in antiphospholipid syndrome and systemic lupus erythematosus [63]. Monocyte transfections with pre-miR-124a and/or -125a caused reduction in target molecules associated with atherothrombosis. Elevated expression of miR-146a/b, miR-21, miR-34a and miR-210 was observed in atherosclerotic plaques compare to non-atherosclerotic left internal thoracic arteries [68]. Patients with stenosis showed an increase in circulating miRNA-21, miRNA-126-3p and miRNA-222 in response to cardiac stress [87]. Finally, miRNA evaluation of 8 patients with stable coronary artery disease display elevated level of miR-145 and reduced expression of miR-126, miR-17, miR-92a and miR-155 compared with healthy controls [67]. The discovery of the role of these miRNAs in arterial thrombosis may provide novel treatment options for cardiovascular diseases.

### 3.3. Coagulation Cascade and Thrombosis 

While several studies suggest that miRNA upregulation results in reduced levels of coagulation factors, numerus studies have also shown that down-regulation of the same miRNAs results in increased expression of these coagulation factors which may trigger thrombotic events.

In addition, mutations have been reported in the 3′UTR of the coagulation factor genes *F2*, *F8*, *F9* and *F11*. Genotyping of Canadian subjects (4485 cases of individuals with extreme low or high level of coagulation factors and 4889 controls) showed an association between some mutations in the 3′UTR of the *F2*, *F8* and *F11* genes with increased plasma activity of the gene-product [16]. In silico studies predicted that these mutations may disturb the binding sites of some miRNAs. Specifically, a mutation within the 3′UTR of *F8* impeded the binding of miR-34a/c and miR-449a/b, while 6 mutations in *F11* 3′UTR impeded the binding of 9 miRNAs which included miR-554 [76].

In another study, reduced level of miR-145 observed in venous thrombosis patients was correlated with an increase in the level of tissue factor [27]. Experimental validation confirmed direct binding of miR-145 with the 3′UTR of the *TF* gene. 

Moreover, it has been suggested that the process of atherosclerotic plaque rupture may be controlled by miR-223-mediated suppression of TF. Overexpression or suppression of miR-223 in HUVEC cells and in mice resulted in dysregulation of tissue factor at both the mRNA and protein levels [30]. It has also been shown that miR-223 is released by exosomes of thrombin activated platelets and is critical for the regulation of atherosclerosis and endothelial implementation. The study showed that miR-223 regulated intercellular adhesion molecule 1 (ICAM-1) expression in thrombin-activated platelet-derived exosomes through NF-κB and MAPK pathways [69]. It has also been demonstrated that some miRNAs released by platelets, including miR-320b can regulate ICAM-1 expression in endothelial cells [88]. 

Studies have also shown that miR-27a/b and miR-494 directly target 3′UTR of tissue factor pathway inhibitor and down-regulate TFPI mRNA and protein levels in MCF7 cells (the human mammary adenocarcinoma cells). Overexpression of these miRNAs induced a procoagulant state caused by increased factor Xa generation which promote thrombotic disease [31].

Finally, polymorphisms in the fibrinogen alpha (FGA) gene have been associated with venous thromboembolism [17,18]. A 28 bp (Del/Ins) in the 3′UTR of the FGA gene was investigated for association with miRNAs. In silico studies identified the binding site of miR-759 within this FGA Del/Ins polymorphic fragment [89]. Studies in a hepatocellular carcinoma cell line HepG2 that endogenously expresses FGA revealed that miR-759 regulates FGA expression. 

The synthesis of fibrinogen increases in response to inflammatory reaction triggered by interleukin-6 (IL-6) [90]. Interestingly, miR-18a has been shown to enhance the IL-6 mediated production of fibrinogen as well as hepatoglobin in human hepatocytes [12].

Recent studies suggest that the decrease in fibrinogen levels may be associated with miRNA-mediated regulation. Transfection of Huh7 hepatoma cells with human miRNAs identified 50 miRNAs that disrupted fibrinogen production in Huh7 cells; of these 50 miRNAs, 27 miRNAs are expressed in the human liver [14]. Among the 27 miRNAs that are expressed in the liver and regulate FI levels; 23 miRNAs downregulated while 4 miRNAs upregulated FI expression. Further studies using the luciferase assay showed that 3 miRNAs (miR-29a, miR-29b, miR-29c; all belonging to the miRNA 29 family) regulate FI expression but do not directly bind to the 3′UTR of FI. On the other hand, miR-409 regulated FI expression by direct binding to fibrinogen Bβ 3′UTR. 

Finally, an evaluation of blood samples from 123 sepsis patient that were divided into coagulation abnormal and normal patients found a correlation between upregulation of miR-122 in coagulation abnormal patients and increased levels of fibrinogen compared to the control group [13].

In addition, coagulation factors there are other proteins involved in hemostasis that can be regulated by miRNAs [91]. Examination of hemostasis-associated genes by RNA pull-down approach revealed 150 specific miRNA associations with hemostatic genes comprising procoagulant (*F7*, *F8*, *F11*, *FGA*, *FGG* and *KLKB1*) and anticoagulant (*SERPINA10*, *PROZ*, *SERPIND1* and *SERPINC1*) as well as fibrinolytic (*PLG*) components. A total of 40 miRNAs were functionally confirmed to target these genes [15].

Genetic Analysis of Idiopathic Thrombophilia 2 using plasma samples from 935 subjects revealed 4 miRNAs (miR-126-3p, miR-885-5p, miR-194-5p and miR-192-5p) that were potentially associated with venous thrombosis; i.e., these miRNAs by targeting genes encoding proteins in the coagulation pathway. miR-885-5p and miR-195-5p expression was correlated with expression of Protein S and FVII. MiR-192-5p dysregulation was associated with FVII and ADAMS13, while miR-126-3p was associated with FXI [20]. Deficiency in protein S can trigger thrombosis [25,92,93,94]. miR-494 has been shown to directly bind to the 3′UTR and downregulate *PROS1* (encoding the protein S) and protein S (PS) levels in liver Huh cells [25].

Finally, a recent study showed that Factor X (FX) deficiency has been associated with dysregulation of miR-24. The evaluation of plasma from 15 healthy volunteers and 36 severe trauma-induced coagulopathy (TIC) patients showed that TIC patients had elevated levels of miR-24, which correlated with significantly lower levels of FX compared to the healthy volunteers [23].

## 4. Discussion

**Figure 1:**
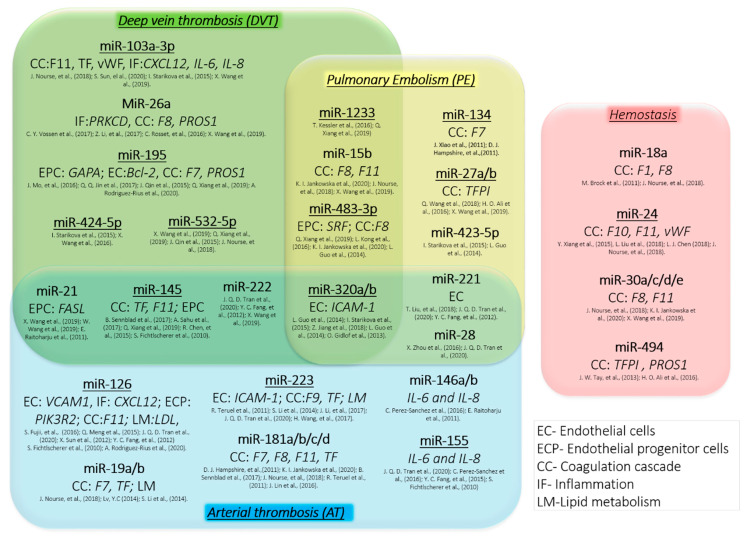
Role of miRNAs in thrombosis and hemostasis. The association of some miRNAs with different types of thrombosis and hemostasis along with the potential target genes (in italic) of proteins essential for coagulation cascade (CC), endothelial progenitor cell (EPC) or endothelial cell (EC) regulation, inflammation (IF) and lipid metabolism (LM). Selected miRNAs have been reported to play an important role in regulation of hemostasis in multiple independent research studies and thus are promising biomarkers (underlined) and therapeutic targets for the treatment of thrombosis and bleeding disorders.

The current studies have highlighted some miRNAs that are essential in thrombosis and hemostasis. As shown in Figure 1, numerous reports indicate a clear association between dysregulation of miR-145, miR-320a/b and miR-222 on thrombosis. Moreover, some miRNAs are linked to specific types of thrombosis, and may thus serve as potential biomarkers or therapeutic targets. For example, miRNAs strongly corelated with deep vein thrombosis such as miR-103a, miR-26 or miR-195 dysregulate protein critical in coagulation factors or target proteins essential for endothelial progenitor cells. Pulmonary embolism is linked with dysregulation of miR27a/b that target tissue factor inhibitor. On other hand, miR-126 or miR-223 that are linked to arterial thrombosis target proteins that cause endothelial dysfunction, alternation of lipid metabolism or trigger inflammation and target ICAM-1, VCAM-1, IL-6 or IL-8. In addition, four miRNAs: miR-18, miR-24, miR-30a/c/d/e and miR-494 has been shown to dysregulate key proteins involved in hemostasis. While these miRNAs have not been linked with thrombosis, they are associated with bleeding disorders. Bleeding disorders and thrombosis can both result from disturbed hemostasis; i.e., decreased and enhanced expression of coagulation proteins respectively. Consequently, we may expect that the reverse dysregulation of these miRNAs may trigger thrombotic event.

Both bleeding disorders and thrombotic events are potentially life-threatening. Both disorders arise due to a disturbance in the homeostasis of the coagulation system and involve many of the same molecular actors. Thus, miRNAs, whose principal biological function is fine-tuning of gene expression, are likely candidates for maintaining the balance between preventing both excessive bleeding and clots that could lead to thrombosis. In the last decade, many research studies have either directly or indirectly implicated miRNAs in regulating the expression of molecules involved in coagulation and/or thrombosis. In this review we have comprehensively and critically surveyed this literature.

The most common bleeding disorders are hemophilia A, B, C and VWD. While these diseases are commonly regarded as being caused by mutations in the coding regions of *F8*, *F9*, *F11* or *VWF*; a critical mass of research studies shows that expression of the protein products of these genes are also regulated by miRNAs. Furthermore, in the case of both hemophilia A and B there are a small group of patients (< 5%) who have no mutations in the coding and non-coding regions of the *F8 and F9* genes. In the case of hemophilia A it has been demonstrated that miRNA dysregulation is a likely cause of the disease in these patients. It is however very likely that in many (possibly most) patients the reduced activity of the coagulation factors is some combination of dysfunction of the protein and reduced expression of the gene. This hypothesis is bolstered by the finding that different patients with the same missense *F8* mutation may exhibit mild, moderate and severe forms of the disease [34] based on FVIII activity. 

Using either in silico or experimental methods (and often both together), a diverse set of miRNAs have been found to associate with the 3′UTRs of genes encoding coagulation factors. This is consistent with the mechanism of action of miRNAs where multiple mRNAs target the same gene and a single miRNA targets multiple genes. It is thus plausible that miRNA dysregulation may be personalized in individual patients and no single miRNA may be associated with the disease condition. Consequently, studies should endeavor to carefully and comprehensively validate the miRNAs associated with disease pathologies but there should be no expectation of consistency between different patients or between different studies. 

We also reviewed the reported role(s) of miRNAs in thrombosis. Venous thromboembolism, deep vein thrombosis, pulmonary embolisms and arterial thrombosis have all been associated with dysregulated miRNAs. 

As expected, many of these miRNAs target genes encoding coagulation factors as up-regulation of proteins involved in hemostasis to abnormally high-levels can lead to thrombotic events. In addition, many other genes not directly involved in the coagulation cascade have also been reported. However, in addition to genetic association studies, more detailed studies that clearly demonstrate the mechanism by which miRNAs physiologically regulate the hemostatic potential are urgently needed. Worldwide, one in four deaths is attributable to thrombosis. Understanding the role of miRNAs in thrombosis represents an untapped potential in the management and treatment of this fatal or debilitating disease.

## Figures and Tables

**Table 1:** MicroRNAs (MiRNAs) that May Target Protein Involved in Coagulation Cascade Which Dysregulation May Lead to Bleeding Disorders or Thrombosis.

Target Gene	miRNA	References
*F1*	miR-18a	[12]
	miR-211	[13]
	miR-218	[14]
	miR-29a/b/c	[14]
	miR-365	[14]
*F1 (FGA)*	miR-193b-3p	[15]
	miR-194-5p	[15]
F1(FGA) *	miR-186	[16]
	miR-3133	[16]
	miR-3173	[16]
	miR-434	[16]
	miR-759	[17,18]
	miR-4476	[16]
*F1 (FGB)*	miR-409-3p	[14]
F1(FGB) *	miR-29b-1-5p	[16]
	miR-4294	[16]
	miR-627	[16]
	miR-759	[16]
	miR-924	[16]
*F1(FGG_A)*	miR-151a-5p	[15]
	miR-193-5p	[15]
	miR-452-5p	[15]
	miR-99b-3p	[15]
*F7*	miR-134 and	[19]
	miR-181a	[19]
	miR-195-5p	[20]
	miR-19a/b-3p	[15]
	MiR-885-5p	[20]
*F8*	let-7i-5p	[8]
	miR-1246,	[9]
	miR-128-3p	[8]
	miR-144-5p	[8]
	miR-15b-3p	[8]
	miR-181d	[9]
	miR-18a-5p	[15]
	miR-30c	[8]
	miR-30e-3p	[15]
	miR-34-5p	[15]
	miR-374b	[8]
	miR-4521	[9]
	miR-454-3p	[15]
	miR-483-3p	[8]
	miR-532-5p	[15]
	miR-6803-3p,	[8]
	miR-7-5p	[15]
	miR-874-3p	[15]
	miR-1297	[21]
F8 *	miR-26a-5p	[21]
	miR-26b-5p	[21]
	miR-34a/c	[16]
	miR-449a/b	[16]
*F9*	miR-128	[22]
	miR-125	[22]
*F10*	miR-24	[23]
*F11*	miR-103a-3p	[15]
	miR-1255a	[15]
	miR-145	[24]
	miR-148b-3p	[15]
	miR-151a-3p	[15]
	miR-15b-5p	[15]
	miR-181a	[24]
	miR-181b-5p	[15]
	miR-24-3p	[15]
	miR-30a-3p	[15]
	miR-30d-3p	[15]
	miR-96-5p	[15]
	miR-126-3p	[20]
F11 *	miR-137	[16]
	miR-1975	[16]
	miR2355	[16]
	miR-4286	[16]
	miR-513a-3p	[16]
	miR-544	[16]
	miR-622	[16]
	miR-889	[16]
	miR-93-5p	[16]
*PROS1*	miR-195-5p	[20]
	MiR-494	[25]
	MiR-885-5p	[20]
PROS1 *	miR-26-5p	[16]
	miR-375	[16]
*TF*	miR-103-3p	[26]
	miR-145	[27]
	miR-181b	[28]
	miR-19b	[29]
	miR-223	[30]
*TFPI*	miR-27a/b	[31]
	miR-494	[31]
TFPI *	miR-200a/b	[16]
	miR-2355	[16]
	miR-429	[16]
	miR-4302	[16]
	miR-605	[16]
*VWF*	miR-24	[32,33]
	miR-103-3p	[26]
*ADAMS13*	MiR-192-5p	[20]

* Gene’s polymorphism

**Table 2:** MiRNAs Associated with Different Types of Thrombosis.

	miRNA	Target Gene	References
DVT	miR-103a-3p	CXCL12	[26]
	miR-126	PIK3R2	[41]
	miR-136-5p		[42]
	miR-150	SRCIN1	[43]
	miR-195	Bcl-2	[44]
		GAPA	[45]
			[46]
	miR-205	PTEN	[47]
	miR-21	FASL	[48]
	MiR-26a	PRKCD	[49]
	miR-320a/b		[50]
	miR-338-5		[51]
	miR-424-5p		[42]
	miR-483-3p	SRF	[52]
	miR-532		[46]
	miR-582		[46]
PE	let-17b	ET-1, TGFBR1	[53]
	miR-106b		[53]
	miR-1233		[54]
	miR-1260		[53]
	miR129-5p		[53]
	miR-134		[55]
	miR-140-3p		[53]
	miR-185		[53]
	miR-1908		[53]
	miR-22		[53]
	miR-221		[56]
	miR-27a/b		[57]
	miR-28		[58]
	miR-320a/b/c		[53]
	miR-423-5p		[53]
	miR-483-5p		[53]
	miR-486		[53]
	miR-602		[53]
	miR-93		[53]
	miR-933		[53]
VTE	miR-103a-3p		[59]
			[60]
	miR-106a-5p		[60]
	miR-10b-5p		[59]
	miR-145		[61]
	miR-15b-5p		[60]
	miR-191-5p		[59]
	miR-195		[61]
	miR-197-3p		[60]
	miR-199b-3p		[59]
	miR-21-5p		[60]
	miR-222-3p		[60]
	miR-26b-5p		[60]
	miR-27b-3p		[60]
	miR-301a-3p		[59]
	miR-30c-5p		[60]
	miR-320a/b		[59]
	miR-361-5p		[60]
	miR-423-5p		[59]
	miR-424-5p		[59]
	miR-483-3p		[61]
	miR-532		[61]
	miR-532-5p		[60]
	miR-652-3p		[60]
AT	miR-10a		[62]
	miR-124a		[63]
	miR-125a		[63]
	miR-126	VCAM-1, CXCL12	[64]
			[65]
			[66]
			[62]
			[67]
	miR-146a		[63]
			[68]
	miR-155		[65]
			[63]
			[62]
			[67]
	miR-17		[67]
	miR-21		[68]
	miR-210		[68]
	miR-221		[65]
			[62]
	miR-222		[65]
			[62]
	miR-223	ICAM-1	[69]
			[62]
			[13]
	miR-28		[62]
	miR-320b	ICAM-1	[27]
	miR-34a		[68]
	miR-431		[62]
	miR-490		[62]
	miR-9		[62]
	miR-92a		[68]

## Abbreviations

Akt	Protein kinase B
APC	Activated protein C
APC-PCI	Plasma concentrations of activated protein C-inhibitor of protein C
APE	Acute pulmonary embolism
ATP	Adenosine triphosphate
Bcl-2	B-cell lymphoma 2
CAD	Coronary artery disease
CCL	Chemokine C-C motif ligand
CTEPH	Chronic thromboembolic pulmonary hypertension
CXCL12	Chemokine C-X-C motif ligand 12
Del/Ins	Deletion-insertion
DVT	Venous thrombosis that includes both deep vein thrombosis
EC	Endothelial cells
EPC	Endothelial progenitor cells
ET-1	Endothelin-1
F11	Coagulation factor FXI
F2	Coagulation factor II gene
F7	Coagulation factor VII gene
F8	Coagulation factor VIII gene
F9	Coagulation factor IX gene
FasL	Fas ligant (FasL or CD95L or CD178) is a type-II transmembrane protein that belongs to the tumor necrosis factor (TNF) family
FASL	Gene encoded Fas ligand
FGA	Fibrinogen alpha gene
FGB	Fibrinogen beta gene
FGG	Fibrinogen gamma gene
FI	Coagulation factor I, fibrinogen
FII	Prothrombin, coagulation factor II
FIX	Coagulation factor IX
FVII	Coagulation factor VII
FVIII	Coagulation factor VIII
FX	Coagulation factor X
FXI	Coagulation factor XI
GABA	Gamma-aminobutyric acid receptors
GABARAPL1	GABA Type A receptor associated protein like 1
HA	Hemophilia A, coagulation FVIII deficiency
HB	Hemophilia B, coagulation FIX deficiency
HC	Hemophilia C, coagulation FXI deficiency
HepG2	Liver hepatocellular cells
Huh-7	Human liver cell line
HUVEC	Human umbilical vein endothelial cells
ICAM-1	Intercellular adhesion molecule 1
IL-6	Interleukin-6
IL-8	Interleukin-8
KLKB1	gene encoded plasma kallikrein
LDL	Low-density lipoprotein
MAPK	Mitogen-activated protein kinase
MCF7	Breast cancer cell line acronym of Michigan Cancer Foundation-7
miRNA	MicroRNAs
MMP2	Proteins of the matrix metalloproteinase
mRNA	Messenger RNA
NF-κB	Nuclear factor-κB
NMD	Nonsense-mediated mRNA decay
OS	Osteosarcoma
PBMC	Peripheral blood mononuclear cells
PE	Pulmonary embolism
PI3K	Phosphoinositide 3-kinases
PIK3R2	Phosphoinositide-3-kinase regulatory subunit 2 which affected PI3K/Akt
PLG	Plasminogen, fibrinolytic protease
PROS	Protein S gene
PROZ	protein Z, vitamin K dependent plasma glycoprotein gene
PS	Protein S
PTC	Premature termination codon
PTEN	Phosphatase and tensin homolog
PTS	Post-thrombotic syndrome
SERPINA	Serpin family A member 1 gene
SERPINC1	Serpin family C member 1 gene
SERPIND1	SERPIND1 gene encoded heparin cofactor II (HCII)
SRC	Proto-oncogene tyrosine-protein kinase Src
SRCIN1	SRC kinase signaling inhibitor 1
SRF	Serum response factor
TGFBR1	Transforming growth factor beta receptor 1
TF	Tissue factor, coagulation factor III
TFPI	Tissue factor pathway inhibitor
TIC	Trauma-induced coagulopathy patients
TNF-α	Tumor necrosis factor alpha
UTR	Untranslated regions
VCAM-1	Vascular cell adhesion molecule-1
VWF	Von Willebrand factor
VWD	Von Willebrand disease
